# Polypharmacy and potentially inappropriate prescribing in people with type 2 diabetes: An analysis of the Scottish Diabetes Research Network national diabetes cohort

**DOI:** 10.1111/dme.70179

**Published:** 2025-11-28

**Authors:** William Berthon, Stuart J. McGurnaghan, Luke A. K. Blackbourn, Amanda de Assuncao Santiago Fernandes, Lauren E. Walker, Rory J. McCrimmon, Helen M. Colhoun, David A. McAllister, Peter Hanlon

**Affiliations:** ^1^ Institute of Genetics and Cancer, College of Medicine and Veterinary Medicine University of Edinburgh Edinburgh UK; ^2^ School of Health and Wellbeing University of Glasgow Glasgow UK; ^3^ Centre for Experimental Therapeutics University of Liverpool Liverpool UK; ^4^ Division of Molecular and Clinical Medicine University of Dundee Dundee UK

**Keywords:** adverse effects of treatment, drug treatment, epidemiology, oral agents, socio‐economic

## Abstract

**Aims:**

This study assesses national trends and, socio‐demographic and clinical factors associated with polypharmacy and potentially in appropriate prescribing among people with type 2 diabetes in Scotland from 2012 to 2022.

**Methods:**

Retrospective cohort study using nationwide data from the Scottish Care Information – Diabetes database. Individuals aged ≥40 years with type 2 diabetes were included. Medication counts were based on unique medications dispensed per calendar year. Potentially inappropriate medications were based on the 2023 Beers criteria and applied to people aged over 65 years. A Poisson mixed‐effects model with individual‐level random intercepts assessed the relationship between the number of drug classes dispensed and year, gender, age group and socio‐economic status, Elixhauser comorbidity index and the hospital frailty risk score.

**Results:**

387,338 people were included. The median number of medications dispensed per person was 9 (interquartile range 5–13). Adjusted medication counts were modestly higher in older people (rate ratio [RR] 1.06, 95% confidence interval [CI] 1.06–1.06 at age 80+ compared to 40–59), higher in women (1.14, 1.13–1.14), in more deprived areas (1.24, 1.23–1.24 in the most deprived vs. the most affluent quintile) and in those with higher comorbidity (1.12, 1.12–1.13 in 4+ vs. 0 comorbidities) but not with high frailty risk (1.00, 1.00–1.00). People over 65 were dispensed a median of 2 (IQR 1–3) potentially inappropriate medications. Potentially inappropriate medication showed a stronger association with comorbidity (1.24, 1.23–1.25) and a positive association with high frailty risk (1.24, 1.23–1.25).

**Conclusions:**

The degree of polypharmacy highlights the need for regular formal medication reviews in this population.

**What‘s new?**

- People with type 2 diabetes in Scotland are prescribed a median of 9 different medications per year.
- People with type 2 diabetes aged over 65 recieve a median of 2 medications per year which are potentially inappropriate.
- Older people, women, people in more deprived areas, and those with comorbidity or frailty and more likely to experience potentially inappropriate prescribing.

## 1 INTRODUCTION

Polypharmacy, the co‐prescription of multiple medications, is common and increasing in the general population and in people with type 2 diabetes.^1^, ^2^, ^3^ For many, this may be appropriate^4^ (e.g. combinations of glucose‐lowering treatments to achieve optimal glycaemic control or optimal management of comorbidities).^5^

However, polypharmacy is associated with risk. Reduced concordance risks reducing the effectiveness of treatment. The likelihood of drug–drug and drug‐disease interactions is proportional to the number of medications that are taken.^1^, ^6^ The number of medications taken and the complexity of regimens also contribute to the workload, or treatment burden, involved in managing long‐term conditions such as type 2 diabetes.^7^, ^8^ People experiencing polypharmacy may also paradoxically be under‐treated for some conditions due to concerns over increasing medication burden and may therefore miss out on potential benefits while still being exposed to the risks of other medications.^9^, ^10^, ^11^

In type 2 diabetes, where polypharmacy is common, it is important to understand the extent of polypharmacy, what types of indications and medications are contributing to this, and the extent to which current levels of polypharmacy may be risky or potentially inappropriate. Therefore, the aim of this study was to quantify polypharmacy and potentially inappropriate prescribing in a nationally comprehensive and representative sample of people with type 2 diabetes in Scotland. We aimed to comprehensively describe polypharmacy via: (i) the number of medications dispensed for each individual; (ii) co‐prescription of medications with similar risks of adverse effects; (iii) the number of potentially inappropriate prescriptions among people aged over 65; and examine how these differed by individual‐level characteristics such as age, sex, socio‐economic status, comorbidity and frailty.

## 2 METHODS

### 2.1 Study design and population

We conducted a cohort study using the Scottish National Diabetes Research Platform.^12^ The Scottish National Diabetes Research Platform contains electronic health records from Scotland's national diabetes information system (SCI‐Diabetes), capturing over 99% of people with diabetes in Scotland. These records were linked to prescription dispensing data from the Scottish National Prescribing Information System, hospital admission records (Scottish Morbidity Record 01, SMR01), mental health admissions (SMR04), cancer registry data (SMR06) and the Scottish Renal Registry. Socio‐economic status was determined using the Scottish Index of Multiple Deprivation (SIMD) 2020 quintiles based on residential postcodes.

The study included all individuals aged 40 years or older with type 2 diabetes who were observable between January 1, 2012 and October 31, 2022. We defined two analytical cohorts: a prevalent cohort to examine overall patterns and an incident cohort to assess changes following diagnosis. Individuals were followed from their study entry date (defined as the latest of: 1 January 2012, their 40th birthday or diabetes diagnosis date) until death, loss of observability or study end.

### 2.2 Medication assessment

Medications were classified using the WHO Anatomical Therapeutic Chemical (ATC) classification system applied to national dispensing records. For each person and for each year, we calculated the number of unique medication classes in use, counting insulin preparations at the ATC‐3 level (pharmacological subgroup) and all other medications at the ATC‐4 level (chemical subgroup) to appropriately distinguish between distinct drug classes while avoiding excessive granularity. We excluded medications typically prescribed for short‐term use (e.g. antibiotics) based on clinical review and validation of prescribing patterns.

### 2.3 Assessment of potentially inappropriate prescribing

For individuals aged ≥65 years, we implemented the 2023 American Geriatrics Society Beers Criteria for potentially inappropriate prescribing^13^ (medications to avoid in older adults, drug‐disease interactions, medications to be used with caution, clinically important drug–drug interactions and medications to avoid or adjust based on kidney function). Each criterion was evaluated using dispensing records supplemented by hospital admission diagnoses and laboratory values where appropriate. Some criteria were adapted or excluded based on the availability of data (e.g. where the appropriateness of a prescription was dependent on the indication or presence of a specific condition, and this could not be reliably identified from the linked data available, we excluded this criterion from the list). A list of all adaptations is available in the supplementary appendix.

We also assessed concurrent use of multiple medications associated with similar adverse drug reactions based on the Scottish Polypharmacy Guideline. We calculated a count of dispensed medications associated with each different risk category.

### 2.4 Covariates

Age was categorised as 40–59, 60–69, 70–79 and ≥80 years. Socio‐economic position was assessed using the Scottish Index of Multiple Deprivation (SIMD), divided into quintiles. Frailty risk was assessed using the hospital frailty risk score and was calculated using ICD‐10 codes from hospital admissions in the 2 years preceding each study year, categorising people as low risk (<5), intermediate risk (5–15) or high risk (>15).^14^ Comorbidity was assessed by a modified Elixhauser comorbidity index using a 10‐year lookback period of hospital admission codes, psychiatric conditions from mental health admissions, cancer diagnoses from the cancer registry and chronic kidney disease from the renal registry. The index was categorised based on unweighted condition counts as 0, 1, 2–3 or ≥4 comorbidities.

### 2.5 Statistical analysis

The distribution of medication counts and (for people aged over 65 years) of the count of potentially inappropriate medications (Beers criteria) was summarised descriptively using medians and interquartile range and stratified by age and sex.

To assess the association between individual characteristics, calendar year and medication use we used mixed‐effects Poisson regression models to analyse medication counts and Beers criteria, with individual‐level random intercepts to account for repeated measures and individual propensity for medication use. For the prevalent cohort, we fitted two models for each outcome: (1) adjusting for calendar year, age group, sex, socio‐economic status and diabetes duration and (2) additionally adjusting for hospital frailty risk score category and Elixhauser comorbidity index. Calendar year was modelled as a categorical variable with 2012 as the reference year. For the incident cohort, we defined years post‐diagnosis as the calendar year following the year of the diagnosis (from +1, the reference level, to +10) and excluded diabetes duration in these models because of its collinearity with the years post‐diagnosis variable. The incident cohort models included the same demographic and clinical covariates as the prevalent cohort models.

All models used follow‐up time weights to account for varying observation periods each year. Results are presented as rate ratios from the reference with 95% confidence intervals. Analyses were conducted using R version 4.1 with the glmmTMB package for modelling.

## 3 RESULTS

### 3.1 Population characteristics and trends

Between 2012 and 2022, the population with type 2 diabetes in Scotland grew from 236,839 to 294,861 individuals, representing a 24.5% increase over the decade (Table 1). The demographic profile remained relatively stable with the mean age increasing slightly from 66.6 (SD 11.8) to 67.5 (SD 11.8) years. The proportion of women decreased from 44.9% to 43.6%. By 2022, 58% (171,089) of the cohort was aged 65 years or older.

**TABLE 1:** Characteristics of people with type 2 diabetes in Scotland, 2012–2022: Cross‐sectional analysis by calendar year in the prevalent cohort.

Calendar year	*N* people	Mean age (SD)	*N* women (%)	Median diabetes duration (IQR)	Median number of dispensed medications per person (IQR)	*N* people aged over 65 (%)	Median number of Beers criteria per person over 65 (IQR)
2012	236,839	66.6 (11.8)	106,343 (44.9%)	6.3 (2.5–11.0)	8 (5–12)	131,541 (55.5%)	2 (1–3)
2013	246,963	66.6 (11.8)	110,269 (44.7%)	6.5 (2.6–11.4)	9 (5–12)	138,592 (56.1%)	2 (1–3)
2014	255,287	66.7 (11.8)	113,531 (44.5%)	6.8 (2.8–11.9)	9 (5–12)	144,167 (56.5%)	2 (1–3)
2015	263,657	66.8 (11.8)	116,701 (44.3%)	7.0 (2.9–12.1)	9 (5–13)	149,677 (56.8%)	2 (1–3)
2016	270,991	66.9 (11.8)	119,486 (44.1%)	7.3 (3.1–12.6)	9 (5–13)	154,110 (56.9%)	2 (1–3)
2017	277,825	67.0 (11.8)	122,164 (44.0%)	7.6 (3.2–13.0)	9 (5–13)	158,500 (57.1%)	2 (1–3)
2018	281,790	67.1 (11.8)	123,364 (43.8%)	7.9 (3.5–13.5)	9 (5–13)	161,214 (57.2%)	2 (1–3)
2019	287,027	67.3 (11.8)	125,351 (43.7%)	8.2 (3.6–13.9)	9 (5–13)	164,832 (57.4%)	2 (1–3)
2020	289,015	67.4 (11.8)	126,027 (43.6%)	8.6 (3.9–14.3)	8 (5–12)	167,008 (57.8%)	2 (1–3)
2021	294,299	67.4 (11.8)	128,341 (43.6%)	8.8 (4.0–14.7)	9 (5–12)	169,822 (57.7%)	2 (1–3)
2022	294,861	67.5 (11.8)	128,608 (43.6%)	9.1 (4.2–15.0)	8 (5–12)	171,089 (58.0%)	2 (1–3)

*Note*: Beers criteria only assessed in individuals aged ≥65 years; Missing values for Beers criteria excluded from calculations; Data available until 31 October 2022.

Abbreviations: IQR, interquartile range (25th–75th percentiles); *N*, number of eligible individuals in each year/time point; SD, standard deviation.

### 3.2 Medication use patterns

**FIGURE 1:**
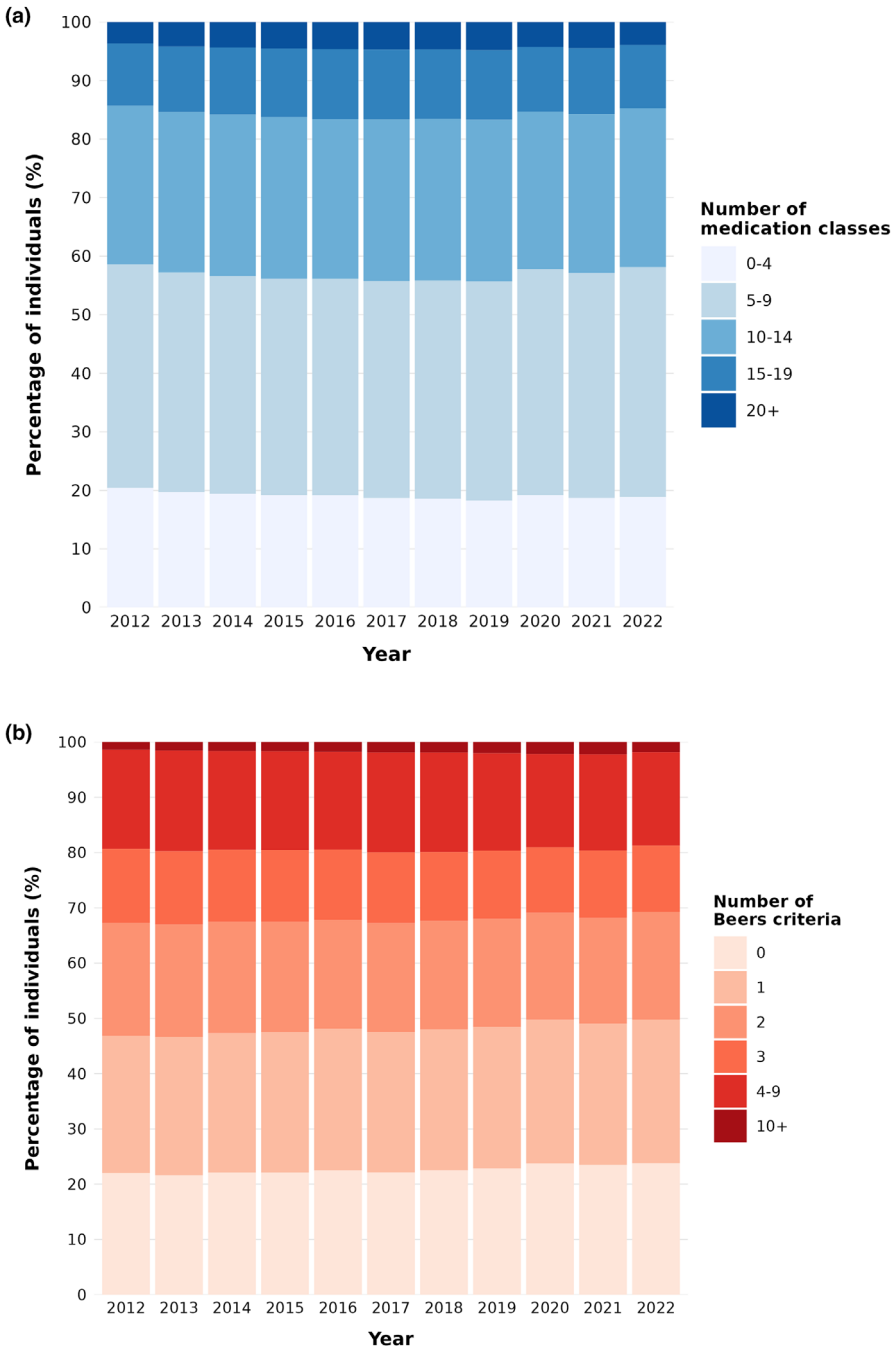
Trends in medication use (a) and potentially inappropriate prescribing (1b) in people with type 2 diabetes Scotland, 2012–2022. This figure shows the distribution of the total number of distinct dispensed medications (panel a) and the total number of Beers criteria met for potentially inappropriate prescribing (panel b) from year 2012 until 2022. Both polypharmacy in general and potentially inappropriate prescribing remained relatively static throughout the study period. Findings are shown here as percentages. The total number of included individuals ranged from 236,839 in 2012 to 294,861 in 2022 (see Table 1).

**FIGURE 2:**
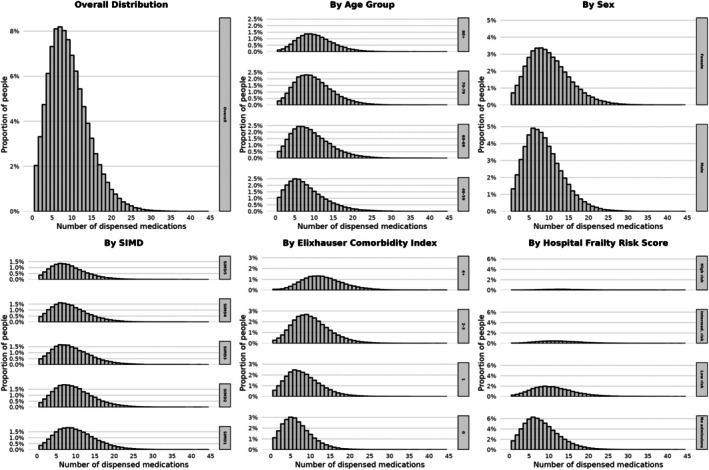
Distribution of medication use by person characteristics and clinical factors in people with type 2 diabetes in Scotland, 2022. These histograms show the distribution of medication counts (defined as the number of distinct medications used per person) in 2022 (*n* = 294,861 people). Findings are presented both overall, and stratified by age, sex, socio‐economic status (SIMD), comorbidity (Elixhauser comorbidity index) and frailty (hospital frailty risk score).

People with type 2 diabetes took a median number of 8–9 distinct medications (IQR 5–12) per person per year throughout the study period (Table 1, Figures 1a and 2). Medication use varied substantially by person characteristics, with higher counts observed in older age groups, women, those from more deprived areas and those with greater clinical complexity (Figure 2). Approximately 40% of people with type 2 diabetes took 10 or more different medications (Figure 1a).

After adjusting for person characteristics, diabetes duration and clinical factors, women were dispensed more medications than men (rate ratio [RR] 1.14 95% CI 1.13–1.14) and while those from the most socio‐economically deprived areas (SIMD1) were dispensed more medications compared to the least deprived (SIMD5) (RR 1.24 95% CI 1.23–1.24) (Table 2). Age was also significantly associated with medication use (RR 1.08 95% CI 1.07–1.08 for age 70–79 compared to those aged 40–59; RR 1.06 95% CI 1.06–1.06 for age 80 and older).

**TABLE 2:** Factors associated with medication counts and potentially inappropriate prescribing in the prevalent cohort, 2012–2022.

Characteristic	Analysis of total number of medications		Analysis of potentially inappropriate medications (Beers criteria) in those aged ≥65	
	Model 1	Model 2	Model 1	Model 2
Age				
40–59	Ref	Ref	–	–
60–69	1.06 (1.05–1.06)***	1.06 (1.05–1.06)***	–	–
70–79	1.08 (1.08–1.09)***	1.08 (1.07–1.08)***	–	–
80+	1.07 (1.07–1.08)***	1.06 (1.06–1.06)***	–	–
Age Beers criteria				
65–69	–	–	Ref	Ref
70–79	–	–	0.99 (0.99–1.00)***	0.99 (0.98–0.99)***
80+	–	–	0.99 (0.99–1.00)	0.97 (0.96–0.97)***
Sex				
Male	Ref	Ref	Ref	Ref
Female	1.14 (1.14–1.15)***	1.14 (1.13–1.14)***	1.29 (1.28–1.30)***	1.28 (1.27–1.29)***
SIMD				
SIMD5 (least deprived)	Ref	Ref	Ref	Ref
SIMD4	1.02 (1.01–1.02)***	1.01 (1.01–1.02)***	1.04 (1.03–1.06)***	1.04 (1.02–1.05)***
SIMD3	1.00 (0.99–1.00)	0.99 (0.99–1.00)*	1.05 (1.03–1.06)***	1.04 (1.02–1.05)***
SIMD2	1.15 (1.14–1.16)***	1.14 (1.14–1.15)***	1.20 (1.18–1.21)***	1.18 (1.16–1.19)***
SIMD1 (most deprived)	1.25 (1.24–1.26)***	1.24 (1.23–1.24)***	1.29 (1.28–1.31)***	1.26 (1.24–1.28)***
Diabetes duration				
Per year post–diagnosis	1.02 (1.02–1.02)***	1.02 (1.02–1.02)***	1.03 (1.03–1.03)***	1.03 (1.03–1.03)***
Elixhauser comorbidity index				
0	–	Ref	–	Ref
1	–	1.04 (1.04–1.04)***	–	1.05 (1.04–1.05)***
2–3	–	1.09 (1.09–1.09)***	–	1.13 (1.12–1.14)***
4+	–	1.12 (1.12–1.13)***	–	1.24 (1.23–1.25)***
Hospital frailty risk score				
No admissions	–	Ref	–	Ref
Low risk	–	1.03 (1.03–1.04)***	–	1.04 (1.04–1.04)***
Intermediate risk	–	1.03 (1.03–1.03)***	–	1.11 (1.11–1.12)***
High risk	–	1.00 (1.00–1.00)	–	1.24 (1.23–1.25)***

*Note*: Values represent Rate Ratios (95% Confidence Intervals); Statistical significance: *p < 0.05; ***p < 0.001. Model 1: Adjusted for diabetes duration and demographic factors (age, sex and socio‐economic status); Model 2: Additionally adjusted for clinical factors (Elixhauser Comorbidity Index and Hospital Frailty Risk Score).

Abbreviations: Ref, reference category; SIMD, Scottish Index of Multiple Deprivation.

### 3.3 Common medications

Table 3 presents the most commonly dispensed medications among people with type 2 diabetes in 2022. Statins were the most frequently dispensed medication class (71.1%), followed by metformin (61.1%) and proton pump inhibitors (47.3%). Cardiovascular medications comprised five of the top ten medication classes with opioids and paracetamol also among the top 10 most commonly dispensed.

**TABLE 3:** Most commonly dispensed medications in people with type 2 diabetes in Scotland in 2022.

Medication (ATC level 4 code)	*N* people (%)
Statins (C10AA)	209,616 (71.1%)
Metformin (A10BA)	180,140 (61.1%)
Proton pump inhibitor (A02BC)	139,387 (47.3%)
ACE inhibitors, plain (C09AA)	116,868 (39.6%)
Platelet aggregation inhibitors excl. heparin (B01AC)	87,590 (29.7%)
Dihydropyridine derivatives (C08CA)	84,181 (28.5%)
Beta blocking agents, selective (C07AB)	76,934 (26.1%)
Opioids in combination with non‐opioid analgesics (N02AJ)	65,979 (22.4%)
Anilides (e.g. Paracetamol) (N02BE)	64,528 (21.9%)
Sulfonylureas (A10BB)	64,014 (21.7%)

When examining specific chemical substances at a lower level, we observed substantial shifts in prescribing patterns between 2012 and 2022 (Table S3). Atorvastatin use increased substantially from 22.5% in 2012 to 43.5% in 2022, while simvastatin use declined from 47.3% to 23.7%. Notably, newer diabetes medications emerged in the top dispensed medications by 2022, with SGLT2 inhibitors (empagliflozin 10.2%, dapagliflozin 6.3%) and GLP‐1 receptor agonists (dulaglutide 3.1%, semaglutide 2.6%) showing rapid uptake. Meanwhile, acetylsalicylic acid use declined markedly (38.1% to 21.0%), likely reflecting changing guidelines on primary prevention of cardiovascular disease.

### 3.4 Potentially inappropriate prescribing (Beers criteria)

**FIGURE 3:**
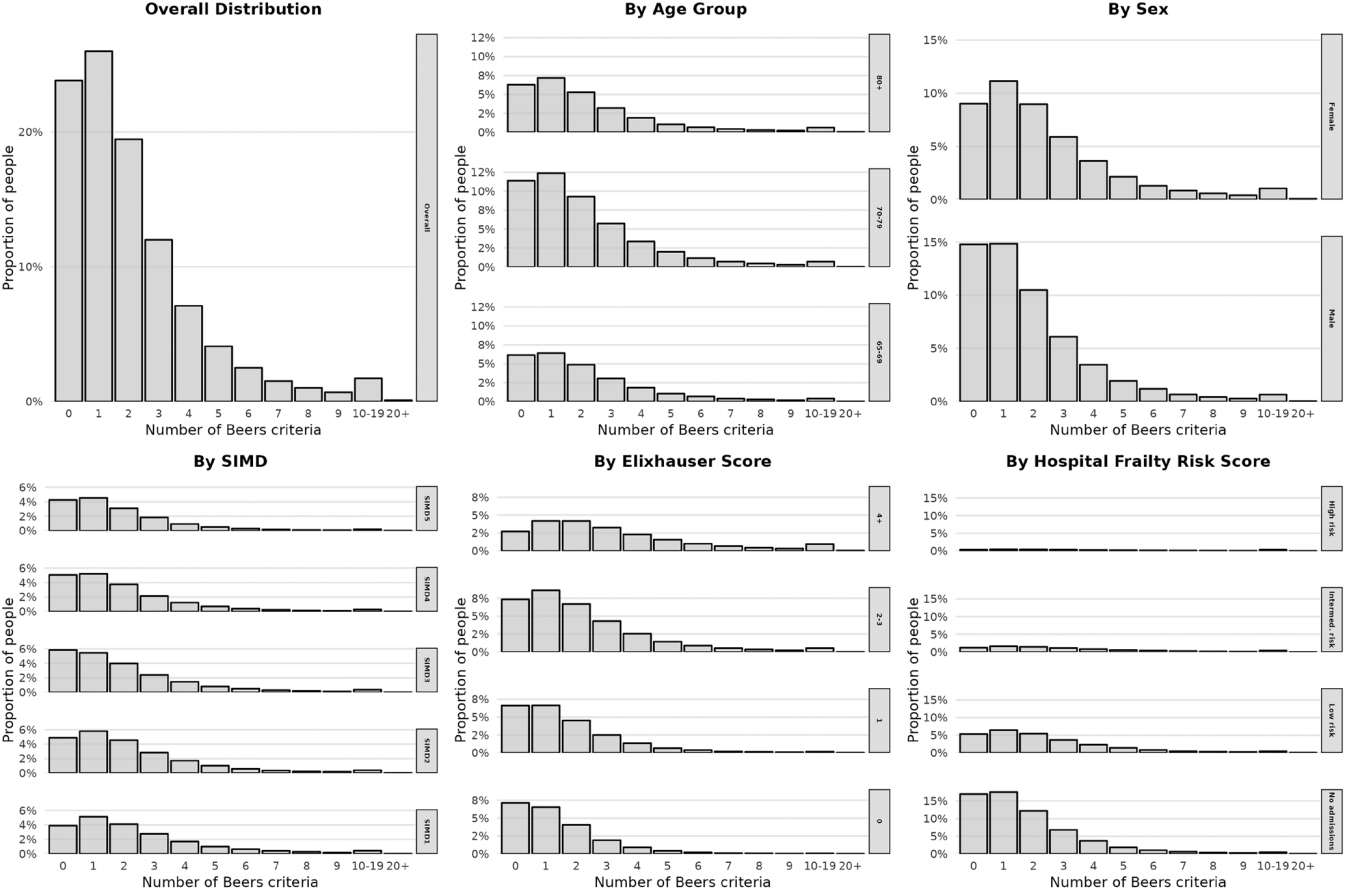
Distribution of potentially inappropriate prescribing by person characteristics and clinical factors in older adults with type 2 diabetes, 2022. These histograms show the distribution of potentially inappropriate prescription counts (defined as the number of distinct medications used per person) in 2022 (*n* = 294,861 people). Findings are presented both overall, and stratified by age, sex, socio‐economic status (SIMD), comorbidity (Elixhauser comorbidity index) and frailty (hospital frailty risk score).

Among individuals aged ≥65 years the median number of Beers criteria remained at 2 (IQR 1–3) throughout the study period, with around 30% of older adults dispensed at least three potentially inappropriate medication indicators (Figure 1b). Figure 3 illustrates the distribution of Beers criteria across person characteristics, showing particularly higher counts in women and those with comorbidity or frailty.

In fully adjusted models, women had higher rates of potentially inappropriate prescribing than men (RR 1.28, 95% CI 1.27–1.29), and those from the most deprived areas had higher rates compared to the least deprived (RR 1.26 95% CI 1.24–1.28). After adjustment for diabetes duration and comorbidity/frailty, older age groups showed slightly lower rates of potentially inappropriate prescribing compared to those aged 65–69 years, with those aged 80+ having fewer Beers criteria indicators (RR 0.97 95% CI 0.96–0.97).

### 3.5 Comorbidity and frailty

Each additional comorbidity was associated with increasing medication counts, with those having 4+ comorbidities experiencing higher medication counts (RR 1.12 95% CI 1.12–1.13) compared to those without comorbidities. While low and intermediate frailty risk were associated with higher medication counts, high‐risk frailty showed no significant association with medication count after adjusting for comorbidities (RR 1.00, 95% CI 1.00–1.00).

High frailty risk was associated with higher rates of Beers criteria indicators (RR 1.24 95% CI 1.23–1.25) and high comorbidity index (Elixhauser score ≥4) had more Beers criteria indicators (RR 1.24 95% CI 1.23–1.25). The strong association between clinical complexity and both medication count and potentially inappropriate prescribing highlights the challenges of medication management in this population.

### 3.6 Cumulative risk from similar drug effects

Analysis of concurrent medications with similar risk profiles is shown in the supplementary appendix (Figures S2–S4). People with type 2 diabetes were frequently prescribed multiple medications increasing the risk of falls and fractures (median of 3 (IQR 2–5) medications per person) constipation (median 3, IQR 1–4) and respiratory function, central nervous system depression and cardiovascular events (median 2, IQR 1–3 for all). As shown in Table S4, the majority of people with type 2 diabetes were exposed to medications with similar risk profiles, with 89.5% receiving at least one medication associated with falls and fractures and 88.1% receiving at least one medication associated with constipation.

### 3.7 Temporal trends

After adjusting for person characteristics, diabetes duration and clinical factors, medication use showed modest changes over the study period, with an initial increase from 2013 to 2019 (up to +3.3%), followed by a COVID‐related decline in 2020, returning to near‐baseline levels by 2022 (+0.2%, 95% CI −0.1 to +0.5% compared to 2012) (Figure S1A). For potentially inappropriate prescribing, the fully adjusted model suggested a modest improvement over time (−6.7% between 2012 and 2022, 95% CI −7.4 to −6.0%) (Figure S1B).

### 3.8 Newly diagnosed people

Findings for people with newly diagnosed type 2 diabetes are shown in the supplementary appendix (Table S1, Figures S2 and S3), with medication counts rising from a median of 7 (IQR 4–10) in the first year after diagnosis to 8 (IQR 6–12) by +10 years. Associations with socio‐demographic and clinical characteristics followed a similar pattern to the prevalent cohort, but with stronger associations between polypharmacy and socio‐economic deprivation, comorbidity and frailty (Table S2).

## 4 DISCUSSION

The degree of polypharmacy among people with type 2 diabetes in Scotland was high, with a median of 9 (IQR 5–12) different medications dispensed per person per year after excluding medications intended only for short‐term use. The number of dispensed medications was 14% higher in women than in men and 24% higher in people living in the most deprived compared to the most affluent quintiles. Commonly prescribed drugs reflected diabetes management and control of cardiovascular risk factors, but also high rates of prescribing for pain, mental health disorders and gastro‐protection. Co‐prescription of medications with similar adverse effects was common. Finally, among people aged over 65 years, dispensing of potentially inappropriate medication was common (median two medications per person meeting the Beers criteria) and was associated with older age, female sex, socio‐economic deprivation, higher comorbidity and a higher degree of frailty risk. Population‐level estimates of polypharmacy and potentially inappropriate prescribing were relatively stable over the past 12 years, however, at an individual‐level polypharmacy increased over time following diagnosis of type 2 diabetes.

Some polypharmacy, such as the high levels of glucose lowering, antihypertensive and cholesterol lowering medications, is likely to be appropriate in the context of type 2 diabetes where such interventions are supported by strong evidence. However, we also showed that co‐prescription of multiple medications with similar risks was common. We did not assess the harm associated with these potentially risky patterns of prescribing, and this remains an important area of future research. Such studies need to carefully consider the causal assumptions, potential confounding factors, individual characteristics (such as frailty) that may increase individual risk, and how to reliably identify harms that occur. Our findings also highlight the wide range of potential risks associated with polypharmacy (e.g. falls, sedation, bleeding, kidney injury) – each of which would require carefully planned analyses taking account of these challenges. While this is beyond the scope of our analysis, our findings highlight a range of potential risks that should be examined further.

Our findings reflect those of previous studies demonstrating the high prevalence of polypharmacy in people with type 2 diabetes, although our study has the advantages of more complete population coverage and analysis of a wider range of characteristics. A systematic review identified 9 studies quantifying polypharmacy in people with type 2 diabetes and reported a pooled prevalence of 50% (95% CI 37–63%) of polypharmacy defined as 5 or more medications.^3^ There was substantial heterogeneity in the study populations, definitions of polypharmacy and the methods used to identify and define medication use. The comparatively higher medication counts in our study may in part reflect our methodology, in which we considered all unique agents dispensed over a 12‐month period (excluding those only used short term). This is a standard approach when assessing population‐level medication burden, but can result in higher medication counts than when considering shorter time‐periods.^15^

Few studies have assessed the overall burden of potentially inappropriate prescribing in type 2 diabetes within the community. A scoping review published in 2022 found that of the 190 studies assessing potentially inappropriate prescribing in diabetes, 51 used explicit listed criteria (such as Beers' criteria); however, none of these specifically assessed the community prevalence of potentially inappropriate prescribing in people with diabetes.^16^ This review did identify inpatient and outpatient‐based studies that demonstrate high rates of potentially inappropriate prescribing in people with diabetes, consistent with our findings, albeit in smaller and less representative samples.

There are likely multiple drivers of polypharmacy and potentially inappropriate prescribing. Firstly, clinical guidelines for type 2 diabetes typically recommend a combination of different medications. The most commonly dispensed drugs identified in our study largely reflect this. Second, comorbidity is the norm in type 2 diabetes. Single‐disease guidelines, when used concurrently, typically lead to the cumulative effect of large numbers of medications. This may be appropriate, but can lead to treatment burden.^17^ Third, there may be a lack of appropriate deprescribing of medications that are causing side effects, are no longer effective or no longer fit with patients' treatment priorities.^18^ Finally, some medications may be started to mitigate the risks of, or in response to side effects of, other long‐term medications. This has the potential to lead to prescribing cascades and accumulation of medications that could potentially be better managed by reviewing or changing medications responsible for risks or side effects.

The high levels of polypharmacy, co‐prescription of medications with similar risks and potentially inappropriate polypharmacy among older people highlight the need for careful review to minimize risks while maintaining the benefits of appropriate medication. Our finding that polypharmacy has remained static over the past 12 years, despite increased emphasis on the importance of polypharmacy and medication reviews, emphasises that reducing medication burden at an individual or population level is challenging. Within this period in Scotland, structured medication reviews are increasingly becoming integrated into primary care, with delivery supported by the integration of clinical pharmacists into many primary care teams.^19^, ^20^ However, the evidence supporting structured medication reviews is variable. While many interventions demonstrate improvements in intermediate outcomes such as numbers of medications or indicators of prescribing quality, there is little evidence of benefit on important clinical outcomes such as falls, hospital admissions or mortality.^21^ How to optimally deliver polypharmacy interventions in a way that improves outcomes that are most meaningful to patients is a priority for future research. This may include digital interventions to streamline medication review and decision support, interventions spanning the interface between primary and secondary care where fragmentation can impede effective medication review or deprescribing and interventions informed by behavioural science seeking to overcome clinical inertia.

These interventions need to be grounded on a rich and detailed understanding of patients' experience. Having demonstrated that the degree of polypharmacy is high, there is a need to understand the burden this places on individuals. Understanding patient perspectives of polypharmacy in the context of type 2 diabetes is important, particularly as the use of multiple concurrent medications is increasingly a feature of diabetes management and reduced concordance has the potential to undermine the potential benefits of treatment. Similarly, future studies should attempt to quantify adherence in the context of polypharmacy, particularly to evidence‐based treatments known to improve outcomes, as suboptimal adherence to potentially beneficial treatments is another possible mechanism by which polypharmacy may lead to harm.

Another priority for future research, as highlighted above, would be to estimate the actual rates of harm caused by potentially inappropriate medication. A few previous studies have assessed associations between polypharmacy and adverse outcomes in type 2 diabetes, showing that polypharmacy in type 2 diabetes is associated with higher all‐cause mortality and cardiovascular events.^22^ However, such analyses are prone to a range of potential biases including confounding by indication and reverse causality.^23^ Our findings indicate that common prescribing patterns in type 2 diabetes span a wide range of potential risks and also that potentially inappropriate prescribing was greatest among people with high frailty risk (who, by definition, are most at risk of physiological decompensation when experiencing adverse events).^24^ Future analyses should focus on specific events (with careful attention to potential confounders) of high clinical impact and on identifying individual characteristics that indicate the greatest risk. These may inform targeted deprescribing to reduce risk.

A key limitation in our assessment was the inability to definitively determine prescribing indications for certain medications. Some medications, even if included within the Beers criteria, may be appropriate in specific circumstances, for certain indications or when alternative approaches are not possible or have been ineffective. While we could identify medication use patterns, the underlying clinical decisions and specific indications were not available in our data. For this reason, we had to adapt our application of the Beers criteria where this was reliant on specific indications. However, as we largely excluded items of the Beers criteria where a lack of indication data would lead to overestimation, our estimate of potentially inappropriate medications is likely to be conservative. Additionally, our assessment of some comorbidities was limited by the availability of historical data, particularly for conditions managed primarily in primary care. The use of hospital admission data for condition identification may have led to underestimation of conditions not typically requiring hospitalisation. Another limitation was that our data included reliable dispensing information only up to October 31, 2022 at the time of preparing this analysis. However, our drug era approach, which spans multiple calendar periods based on dispensing patterns, helps mitigate the impact of this truncation on the final study year's raw counts, and the use of follow‐up time weights in the models accounts for varying observation periods within each year to generate the estimates. Additionally, the size of the dataset (with 2,998,554 person‐years and often multiple medications per person‐year) necessitated analysis at the annual rather than a more granular level due to computational constraints.

## 5 CONCLUSION

Among people with type 2 diabetes in Scotland, the degree of polypharmacy and potentially inappropriate medication use has remained high over the past 12 years. Older people, women, people living in more deprived areas and those with high comorbidity or risk of frailty experience the greatest degree of polypharmacy and the highest burden of potentially inappropriate medication. While the use of multiple medications remains an important aspect of diabetes management, our findings emphasise the need to quantify and understand the risk associated with polypharmacy, better understand patients' perspectives on polypharmacy and the need for evidence‐based interventions to support medication optimization.

## AUTHOR CONTRIBUTIONS

PH, DM and HC conceived the study. PH, DM and HC wrote the analysis plan. AdASF and PH adapted the Beers criteria definitions for application to the dataset. WB performed the analysis with support from SM and LB. All authors reviewed the analysis results and interpreted the results. WB and PH wrote the first draft of the manuscript. All authors reviewed this and subsequent drafts and contributed content. All authors approved the final draft.

## FUNDING INFORMATION

This study was funded by Diabetes UK and Pharmacy Research UK (Grant reference: 23/0006612).

## CONFLICT OF INTEREST STATEMENT

The authors declare no conflicts of interest.

## Supporting information


Appendix S1.
