# Supplementary material for: Polypharmacy and potentially inappropriate prescribing in people with type 2 diabetes: An analysis of the Scottish Diabetes Research Network national diabetes cohort
Source: Diabet Med. 2025 Nov 28;43(2):e70179. doi: 10.1111/dme.70179 (PMC12857861; doi:10.1111/dme.70179)
Supplement: Supplementary file 1 — Appendix S1. [file DME-43-e70179-s001.docx]

**SUPPLEMENTARY METHODS**

**Detailed Cohort Construction**

We defined two analytical cohorts from the Scottish Diabetes Research Network national diabetes dataset. The prevalent cohort included anyone with type 2 diabetes who was aged 40 years or older during 2012-2022, regardless of their age at diagnosis. For the incident cohort, we identified individuals newly diagnosed with type 2 diabetes at age ≥40 years between 2012-2022. Person-time was calculated on a yearly basis, with individuals contributing to each calendar year in which they were observable. Observability was determined using a combination of prescription dispensing records and routine clinical measurements. For the incident cohort, we excluded the calendar year of diagnosis to ensure complete medication capture, counting years post-diagnosis by calendar year and beginning follow-up from the first calendar year after diagnosis (year +1). This approach maintained consistency in medication assessment across the cohort.

**Drug Era Construction and Medication Assessment**

We constructed continuous drug eras using prescription dispensing records with a 56-day bridging period between dispensings, selected to align with the standard prescription duration in Scottish clinical practice. A drug era began on the first dispensing date and continued until a gap of 56 days occurred between the projected end date of one prescription and the start of the next. This approach accounts for early refills and variable prescription durations common in clinical practice.

For each calendar year, medications were included in counts if their drug era overlapped with that year at any point. For partially observable years (e.g., due to death, loss to follow-up, or the end of data availability in October 2022), we adjusted for observation time in our statistical models using follow-up time weights.

Medications were excluded from continuous counts if classified as short-term based on their typical duration of use and prescribing patterns. This classification, developed through clinical review, identified antimicrobials (antibiotics, antivirals, antifungals), vaccines, and medications typically prescribed for acute conditions. While these medications were excluded from continuous medication counts, they were retained in analyses of potentially inappropriate prescribing to capture important drug-drug interactions.

**Implementation of Beers Criteria, Potentially Inappropriate Prescribing**

The 2023 Beers Criteria were implemented using a hierarchical approach to medication matching. ATC codes in the criteria were matched at multiple levels (ATC-2 through ATC-5) to ensure complete capture of medication classes while maintaining specificity for individual drugs. For medications requiring assessment of duration (e.g., proton pump inhibitors, nitrofurantoin), we calculated both the total duration of the drug era and the cumulative exposure within each calendar year.

Long-term use was defined as drug era durations exceeding 112 days, which effectively captures more than two prescriptions within a 12-month period given standard 56-day prescription durations in Scotland. This approach was validated against raw dispensing counts and showed good concordance for medications requiring duration assessment.

Drug-disease interactions were identified using specific lookback periods for each condition:

- Falls and syncope: Two-year lookback from each prescription date
- Heart failure, dementia, Parkinson's disease: Any prior occurrence

For lower urinary tract symptoms (LUTS) or benign prostatic hyperplasia (BPH) in men, we identified the condition through any use of alpha-adrenergic antagonists (G04CA) or 5-alpha reductase inhibitors (G04CB). The earliest dispensing date of either medication class established the condition date, after which any contraindicated medications were flagged as potentially inappropriate. This pharmaceutical-based approach to condition identification, while highly specific, was limited to the period from which electronic prescribing data was available (2009 onwards).

Drug-drug interactions were assessed using a combination of direct paired analysis for specific drug combinations and a broader assessment of central nervous system-active medications, where concurrent use of three or more such medications was flagged as potentially inappropriate.

For estrogen preparations, we included both single-ingredient products (G03C) and estrogen-progestogen combinations (G03F), excluding topical preparations. NSAIDs were assessed comprehensively, including all products and combinations. For antipsychotic medications, lithium (N05AN) was excluded from the analysis as it has distinct prescribing indications and monitoring requirements.

For medications requiring dose adjustment based on kidney function (Beers Table 6), we used estimated glomerular filtration rate (eGFR), as this was available for most people in our electronic health records. While the original Beers criteria specify creatinine clearance (CrCl), we applied conventional eGFR thresholds (60, 30, 15 mL/min/1.73m²) following expert consultation. This approach, while a pragmatic compromise, enables comprehensive assessment of potentially inappropriate prescribing while maintaining consistency with clinical practice where eGFR is the routinely available measure of kidney function.

**Implementation of Clinical Complexity Measures**

The Hospital Frailty Risk Score was calculated for each study year using hospital admissions from the preceding two years. We updated the original scoring algorithm to include additional ICD-10 codes that had been introduced since the score's development, maintaining consistency with the original weighting scheme. Individual scores were categorised as low risk (<5), intermediate risk (5-15), or high risk (>15) based on established thresholds. The modified codes and their weights are provided in Supplementary Table S1.

For the Elixhauser Comorbidity Index, we implemented a comprehensive capture of conditions using multiple data sources. The primary source was hospital admission records with a 10-year lookback period, supplemented by psychiatric conditions from mental health admissions (SMR04) and cancer diagnoses from the cancer registry (SMR06), with the exception of renal failure where the entire available history was considered using information from the Scottish Renal Registry. When conditions appeared in multiple sources, we reconciled dates to ensure accurate temporal assessment. The final index was categorised based on unweighted condition counts as 0 (no comorbidities), 1 (single comorbidity), 2-3 comorbidities, or ≥4 comorbidities.

**Statistical Analysis Technical Details**

Mixed-effects Poisson regression models were fitted using the glmmTMB package in R, with optimisation performed using the BFGS algorithm. For the incident cohort models by calendar year, we used the nlminb optimiser with extended iteration limits (2000) to resolve some convergence issues. Initial analyses considered both Poisson and negative binomial distributions, but the inclusion of random intercepts for each individual adequately accounted for overdispersion by allowing individual-specific baseline medication levels. This approach effectively captured the consistent differences between individuals in their medication use patterns while maintaining model interpretability. Model diagnostics, including examination of random effects distributions and comparison of observed versus predicted counts, confirmed appropriate model fit.

To examine potential interactions between calendar time and diabetes duration, we fitted separate models for each calendar year, with years post-diagnosis included as a categorical factor. We started from 2014 to ensure sufficient variation in the years post-diagnosis variable, as 2013 data would contain only individuals with 1 year post-diagnosis (diagnosed in 2012). This approach allowed us to visualise how the relationship between disease duration and medication use varied across different calendar periods.

**SUPPLEMENTARY TABLES**

*Table S1. Characteristics of people with type 2 diabetes in Scotland, 2012-2022: Longitudinal analysis post-year of diabetes diagnosis in people diagnosed from 2012 onwards (incident cohort)*

| **Calendar year following diagnosis** | **N people** | **Mean age (SD)** | **N females (%)** | **Median number of dispensed medications per person (IQR)** | **N people aged over 65 (%)** | **Median number of Beers criteria per person over 65 (IQR)** |
| --- | --- | --- | --- | --- | --- | --- |
| +1 year | 159,733 | 62.4 (11.4) | 68,537 (42.9%) | 7 (4-10) | 64,349 (40.3%) | 1 (0-2) |
| +2 years | 139,811 | 63.2 (11.3) | 59,786 (42.8%) | 7 (4-11) | 59,894 (42.8%) | 1 (0-2) |
| +3 years | 124,701 | 64.1 (11.2) | 53,355 (42.8%) | 7 (4-11) | 56,619 (45.4%) | 1 (0-2) |
| +4 years | 107,641 | 64.8 (11.0) | 46,014 (42.7%) | 7 (4-11) | 51,528 (47.9%) | 1 (0-2) |
| +5 years | 92,143 | 65.6 (10.9) | 39,508 (42.9%) | 8 (5-11) | 46,527 (50.5%) | 1 (0-2) |
| +6 years | 75,258 | 66.3 (10.8) | 32,288 (42.9%) | 8 (5-11) | 39,665 (52.7%) | 1 (0-3) |
| +7 years | 58,810 | 66.9 (10.7) | 25,176 (42.8%) | 8 (5-12) | 32,286 (54.9%) | 1 (0-3) |
| +8 years | 42,775 | 67.5 (10.5) | 18,251 (42.7%) | 8 (5-12) | 24,421 (57.1%) | 1 (0-3) |
| +9 years | 28,294 | 68.3 (10.3) | 12,091 (42.7%) | 8 (5-12) | 16,867 (59.6%) | 1 (0-3) |
| +10 years | 13,543 | 68.8 (10.1) | 5,767 (42.6%) | 8 (6-12) | 8,345 (61.6%) | 1 (0-3) |

Notes: N: Number of eligible individuals in each year/time point; SD: Standard deviation; IQR: Interquartile range (25th-75th percentiles); Beers criteria only assessed in individuals aged ≥65 year; Missing values for Beers criteria excluded from calculations; Data available until 31 October 2022.

*Table S2. Factors associated with medication counts and potentially inappropriate prescribing (Beers criteria) in people diagnosed from 2012 onwards (incident cohort)*

| **Characteristic** | **Analysis of total number of medications** | | **Analysis of potentially inappropriate medications (Beers criteria) in those aged ≥65** | |
| --- | --- | --- | --- | --- |
|  | **Model 1** | **Model 2** | **Model 1** | **Model 2** |
| **Age** |  |  |  |  |
| 40-59 | Ref | Ref | - | - |
| 60-69 | 1.08 (1.08-1.08)*** | 1.08 (1.07-1.08)*** | - | - |
| 70-79 | 1.14 (1.13-1.14)*** | 1.12 (1.12-1.13)*** | - | - |
| 80+ | 1.18 (1.17-1.18)*** | 1.14 (1.13-1.15)*** | - | - |
| **Age Beers Criteria** |  |  |  |  |
| 65-69 | - | - | Ref | Ref |
| 70-79 | - | - | 1.01 (1.00-1.02) | 0.99 (0.98-1.00) |
| 80+ | - | - | 1.08 (1.06-1.09)*** | 1.02 (1.01-1.04)** |
| **Sex** |  |  |  |  |
| Male | Ref | Ref | Ref | Ref |
| Female | 1.22 (1.21-1.23)*** | 1.21 (1.20-1.22)*** | 1.47 (1.45-1.49)*** | 1.46 (1.44-1.48)*** |
| **SIMD** |  |  |  |  |
| SIMD5 (least deprived) | Ref | Ref | Ref | Ref |
| SIMD4 | 1.03 (1.02-1.04)*** | 1.03 (1.01-1.04)*** | 1.07 (1.05-1.10)*** | 1.06 (1.04-1.09)*** |
| SIMD3 | 1.01 (1.00-1.02)* | 1.01 (1.00-1.02) | 1.08 (1.05-1.11)*** | 1.07 (1.04-1.10)*** |
| SIMD2 | 1.19 (1.18-1.21)*** | 1.18 (1.17-1.19)*** | 1.27 (1.24-1.31)*** | 1.24 (1.21-1.27)*** |
| SIMD1 (most deprived) | 1.33 (1.31-1.34)*** | 1.30 (1.29-1.32)*** | 1.44 (1.41-1.48)*** | 1.39 (1.35-1.42)*** |
| **Elixhauser Comorbidity Index** |  |  |  |  |
| 0 | - | Ref | - | Ref |
| 1 | - | 1.07 (1.07-1.08)*** | - | 1.10 (1.08-1.11)*** |
| 2-3 | - | 1.17 (1.16-1.17)*** | - | 1.26 (1.24-1.28)*** |
| 4+ | - | 1.25 (1.24-1.25)*** | - | 1.48 (1.45-1.50)*** |
| **Hospital Frailty Risk Score** |  |  |  |  |
| No admissions | - | Ref | - | Ref |
| Low risk | - | 1.03 (1.03-1.04)*** | - | 1.04 (1.04-1.05)*** |
| Intermediate risk | - | 1.04 (1.04-1.05)*** | - | 1.15 (1.14-1.17)*** |
| High risk | - | 1.03 (1.03-1.04)*** | - | 1.29 (1.27-1.32)*** |

Notes: Values represent Rate Ratios (95% Confidence Intervals); Statistical significance: * p<0.05, ** p<0.01, *** p<0.001; Model 1: Adjusted for demographic factors (age, sex, socioeconomic status); Model 2: Additionally adjusted for clinical factors (Elixhauser Comorbidity Index and Hospital Frailty Risk Score); Ref: Reference category; SIMD: Scottish Index of Multiple Deprivation; Analysis includes individuals diagnosed from 2012 onwards, excluding year of diagnosis.

*Table S3. Trends in most commonly dispensed medications (ATC level 5) among people with type 2 diabetes in Scotland in 2012, 2017 and 2022 (ranked by 2022 prevalence, excluding short-term medications)*

| **Medication** | **ATC code** | **2012** | **2017** | **2022** |
| --- | --- | --- | --- | --- |
| Metformin | A10BA02 | 129,003 (54.7%) | 158,374 (57.1%) | 180,140 (61.2%) |
| Atorvastatin | C10AA05 | 53,179 (22.5%) | 89,534 (32.3%) | 127,954 (43.5%) |
| Omeprazole | A02BC01 | 60,560 (25.7%) | 78,063 (28.2%) | 86,801 (29.5%) |
| Amlodipine | C08CA01 | 48,575 (20.6%) | 61,743 (22.3%) | 70,959 (24.1%) |
| Simvastatin | C10AA01 | 111,516 (47.3%) | 97,558 (35.2%) | 69,923 (23.7%) |
| Ramipril | C09AA05 | 50,688 (21.5%) | 62,210 (22.4%) | 69,166 (23.5%) |
| Paracetamol | N02BE01 | 53,233 (22.6%) | 67,032 (24.2%) | 64,528 (21.9%) |
| Acetylsalicylic acid | B01AC06 | 89,993 (38.1%) | 73,604 (26.5%) | 61,888 (21.0%) |
| Codeine and Paracetamol | N02AJ06 | 59,392 (25.2%) | 68,249 (24.6%) | 59,567 (20.2%) |
| Gliclazide | A10BB09 | 55,995 (23.7%) | 63,789 (23.0%) | 58,109 (19.7%) |
| Bisoprolol | C07AB07 | 26,101 (11.1%) | 44,180 (15.9%) | 55,797 (18.9%) |
| Salbutamol | R03AC02 | 34,814 (14.8%) | 47,786 (17.2%) | 50,158 (17.0%) |
| Lansoprazole | A02BC03 | 26,569 (11.3%) | 38,286 (13.8%) | 48,279 (16.4%) |
| Furosemide | C03CA01 | 33,811 (14.3%) | 37,383 (13.5%) | 35,668 (12.1%) |
| Amitriptyline | N06AA09 | 23,702 (10.0%) | 31,154 (11.2%) | 33,545 (11.4%) |
| Lisinopril | C09AA03 | 30,801 (13.1%) | 32,557 (11.7%) | 31,113 (10.6%) |
| Levothyroxine sodium | H03AA01 | 22,445 (9.5%) | 28,384 (10.2%) | 30,635 (10.4%) |
| Ibuprofen | M02AA13 | 7,736 (3.3%) | 31,252 (11.3%) | 30,605 (10.4%) |
| Clopidogrel | B01AC04 | 15,420 (6.5%) | 26,207 (9.5%) | 30,512 (10.4%) |
| Empagliflozin | A10BK03 |  | 7,835 (2.8%) | 30,152 (10.2%) |
| Glyceryl trinitrate | C01DA02 | 27,993 (11.9%) | 33,193 (12.0%) | 29,671 (10.1%) |
| Bendroflumethiazide | C03AA01 | 45,008 (19.1%) | 38,450 (13.9%) | 28,616 (9.7%) |
| Folic acid | B03BB01 | 8,792 (3.7%) | 19,093 (6.9%) | 27,623 (9.4%) |
| Ferrous fumarate | B03AA02 | 15,666 (6.6%) | 21,789 (7.9%) | 24,324 (8.3%) |
| Prednisolone | H02AB06 | 16,335 (6.9%) | 24,161 (8.7%) | 21,522 (7.3%) |
| Tamsulosin | G04CA02 | 10,617 (4.5%) | 16,859 (6.1%) | 21,138 (7.2%) |
| Losartan | C09CA01 | 13,861 (5.9%) | 19,668 (7.1%) | 21,057 (7.2%) |
| Calcium, combinations with Vitamin D and/or other drugs | A12AX | 16,358 (6.9%) | 20,133 (7.3%) | 20,790 (7.1%) |
| Atenolol | C07AB03 | 35,406 (15.0%) | 29,118 (10.5%) | 20,476 (7.0%) |
| Colecalciferol | A11CC05 | 414 (0.2%) | 11,437 (4.1%) | 19,327 (6.6%) |
| Sertraline | N06AB06 | 4,653 (2.0%) | 13,038 (4.7%) | 19,160 (6.5%) |
| Dapagliflozin | A10BK01 |  | 8,342 (3.0%) | 18,587 (6.3%) |
| Tramadol | N02AX02 | 23,893 (10.1%) | 23,374 (8.4%) | 18,026 (6.1%) |
| Naproxen | M01AE02 | 7,918 (3.4%) | 19,914 (7.2%) | 17,658 (6.0%) |
| Alginic acid | A02BX13 | 8,140 (3.4%) | 14,497 (5.2%) | 17,511 (5.9%) |
| Candesartan | C09CA06 | 12,002 (5.1%) | 16,109 (5.8%) | 17,485 (5.9%) |
| Alogliptin | A10BH04 |  | 5,719 (2.1%) | 17,404 (5.9%) |
| Gabapentin | N02BF01 | 11,306 (4.8%) | 19,295 (7.0%) | 17,352 (5.9%) |
| Cetirizine | R06AE07 | 12,357 (5.2%) | 17,080 (6.2%) | 17,173 (5.8%) |
| Allopurinol | M04AA01 | 10,959 (4.6%) | 15,229 (5.5%) | 17,064 (5.8%) |
| Senna glycosides | A06AB06 | 10,026 (4.2%) | 14,123 (5.1%) | 16,359 (5.6%) |
| Clotrimazole | D01AC01 | 9,814 (4.2%) | 14,418 (5.2%) | 16,110 (5.5%) |
| Doxazosin | C02CA04 | 16,119 (6.8%) | 17,180 (6.2%) | 15,970 (5.4%) |
| Lactulose | A06AD11 | 14,627 (6.2%) | 16,915 (6.1%) | 15,721 (5.3%) |
| Isosorbide mononitrate | C01DA14 | 14,170 (6.0%) | 15,290 (5.5%) | 15,395 (5.2%) |
| Imidazoles/Triazoles in combination with Corticosteroids | D01AC20 | 13,073 (5.5%) | 17,997 (6.5%) | 15,322 (5.2%) |
| Hydroxocobalamin | B03BA03 | 8,754 (3.7%) | 14,747 (5.3%) | 15,076 (5.1%) |
| Mirtazapine | N06AX11 | 5,412 (2.3%) | 11,284 (4.1%) | 15,035 (5.1%) |
| Pregabalin | N02BF02 | 4,212 (1.8%) | 9,664 (3.5%) | 13,664 (4.6%) |
| Apixaban | B01AF02 |  | 6,728 (2.4%) | 13,361 (4.5%) |
| Sildenafil | G04BE03 | 10,216 (4.3%) | 15,178 (5.5%) | 12,891 (4.4%) |
| Perindopril | C09AA04 | 14,971 (6.3%) | 14,879 (5.4%) | 12,620 (4.3%) |
| Diazepam | N05BA01 | 11,185 (4.7%) | 14,502 (5.2%) | 12,518 (4.3%) |
| Loperamide | A07DA03 | 10,381 (4.4%) | 12,839 (4.6%) | 12,256 (4.2%) |
| Fluoxetine | N06AB03 | 8,849 (3.7%) | 11,462 (4.1%) | 11,661 (4.0%) |
| Rosuvastatin | C10AA07 | 10,547 (4.5%) | 10,009 (3.6%) | 11,561 (3.9%) |
| Formoterol and Beclometasone | R03AK08 | 635 (0.3%) | 8,307 (3.0%) | 11,529 (3.9%) |
| Morphine | N02AA01 | 5,393 (2.3%) | 9,930 (3.6%) | 11,374 (3.9%) |
| Dihydrocodeine | N02AA08 | 8,079 (3.4%) | 11,334 (4.1%) | 11,247 (3.8%) |
| Sitagliptin | A10BH01 | 14,309 (6.1%) | 20,983 (7.6%) | 11,241 (3.8%) |
| Betamethasone | D07AC01 | 10,545 (4.5%) | 13,740 (5.0%) | 11,228 (3.8%) |
| Diclofenac | M02AA15 | 7,449 (3.2%) | 11,930 (4.3%) | 11,010 (3.7%) |
| Citalopram | N06AB04 | 12,930 (5.5%) | 12,733 (4.6%) | 10,877 (3.7%) |
| Finasteride | G04CB01 | 5,658 (2.4%) | 9,064 (3.3%) | 10,633 (3.6%) |
| Zopiclone | N05CF01 | 8,012 (3.4%) | 12,281 (4.4%) | 10,428 (3.5%) |
| Artificial tears and other indifferent preparations | S01XA20 | 9,764 (4.1%) | 12,065 (4.4%) | 10,249 (3.5%) |
| Beclometasone | R01AD01 | 8,940 (3.8%) | 12,796 (4.6%) | 10,237 (3.5%) |
| Codeine | R05DA04 | 4,850 (2.1%) | 8,736 (3.2%) | 9,888 (3.4%) |
| Beclometasone | R03BA01 | 9,177 (3.9%) | 10,259 (3.7%) | 9,847 (3.3%) |
| Butylscopolamine | A03BB01 | 4,239 (1.8%) | 8,312 (3.0%) | 9,595 (3.3%) |
| Insulin (human) | A10AD01 | 5,091 (2.2%) | 8,424 (3.0%) | 9,504 (3.2%) |
| Prochlorperazine | N05AB04 | 9,287 (3.9%) | 10,818 (3.9%) | 9,177 (3.1%) |
| Dulaglutide | A10BJ05 |  | 2,098 (0.8%) | 9,146 (3.1%) |
| Fexofenadine | R06AX26 | 2,550 (1.1%) | 5,255 (1.9%) | 8,971 (3.0%) |
| Propranolol | C07AA05 | 4,366 (1.8%) | 6,846 (2.5%) | 8,946 (3.0%) |
| Edoxaban | B01AF03 |  | 50 (<0.1%) | 8,612 (2.9%) |
| Indapamide | C03BA11 | 3,854 (1.6%) | 6,997 (2.5%) | 8,540 (2.9%) |
| Mometasone | R01AD09 | 6,567 (2.8%) | 7,523 (2.7%) | 8,536 (2.9%) |
| Insulin glargine | A10AE04 | 7,858 (3.3%) | 8,203 (3.0%) | 8,053 (2.7%) |
| Hydrocortisone | D07AA02 | 6,870 (2.9%) | 9,414 (3.4%) | 7,941 (2.7%) |
| Duloxetine | N06AX21 | 2,589 (1.1%) | 5,491 (2.0%) | 7,858 (2.7%) |
| Solifenacin | G04BD08 | 5,798 (2.5%) | 7,462 (2.7%) | 7,835 (2.7%) |
| Semaglutide | A10BJ06 |  |  | 7,721 (2.6%) |
| Dihydrocodeine and Paracetamol | N02AJ01 | 12,228 (5.2%) | 10,163 (3.7%) | 7,695 (2.6%) |
| Clobetasone | D07AB01 | 7,641 (3.2%) | 9,116 (3.3%) | 7,682 (2.6%) |
| Spironolactone | C03DA01 | 5,292 (2.2%) | 7,021 (2.5%) | 7,463 (2.5%) |
| Lidocaine | N01BB02 | 998 (0.4%) | 3,475 (1.3%) | 7,383 (2.5%) |
| Ibuprofen | M01AE01 | 12,675 (5.4%) | 10,792 (3.9%) | 7,339 (2.5%) |
| Digoxin | C01AA05 | 7,072 (3.0%) | 7,725 (2.8%) | 7,233 (2.5%) |
| Ispaghula (psylla seeds) | A06AC01 | 5,680 (2.4%) | 6,792 (2.4%) | 7,230 (2.5%) |
| Insulin (human) | A10AC01 | 3,100 (1.3%) | 6,541 (2.4%) | 7,222 (2.5%) |
| Esomeprazole | A02BC05 | 3,080 (1.3%) | 5,143 (1.9%) | 7,213 (2.4%) |
| Alendronic acid | M05BA04 | 7,788 (3.3%) | 8,246 (3.0%) | 7,152 (2.4%) |
| Pioglitazone | A10BG03 | 18,167 (7.7%) | 10,900 (3.9%) | 6,977 (2.4%) |
| Felodipine | C08CA02 | 6,823 (2.9%) | 7,342 (2.6%) | 6,880 (2.3%) |
| Cyanocobalamin | B03BA01 | 1,732 (0.7%) | 3,679 (1.3%) | 6,845 (2.3%) |
| Canagliflozin | A10BK02 |  | 2,496 (0.9%) | 6,814 (2.3%) |
| Warfarin | B01AA03 | 14,476 (6.1%) | 14,362 (5.2%) | 6,651 (2.3%) |
| Venlafaxine | N06AX16 | 2,792 (1.2%) | 5,001 (1.8%) | 6,592 (2.2%) |
| Chloramphenicol | S01AA01 | 9,018 (3.8%) | 9,802 (3.5%) | 6,590 (2.2%) |

*Table S4. Medication-related risk exposure in people with type 2 diabetes in Scotland, 2022: Prevalence by adverse effect risk category*

| **Risk Category** | **Number of People Exposed** | **Percent of Population** |
| --- | --- | --- |
| Falls and Fractures | 263,983 | 89.5% |
| Constipation | 259,769 | 88.1% |
| Cardiovascular Events | 243,756 | 82.7% |
| Respiratory | 242,481 | 82.2% |
| Central Nervous System Depression | 223,294 | 75.7% |
| Renal Injury | 197,624 | 67.0% |
| Bleeding | 178,636 | 60.6% |
| Urinary Retention | 168,746 | 57.2% |
| Hyperkalaemia | 165,961 | 56.3% |
| Serotonin Syndrome | 142,634 | 48.4% |
| Hypoglycaemia | 140,204 | 47.5% |
| Heart Failure | 135,133 | 45.8% |
| Hypokalaemia | 104,277 | 35.4% |
| Bradycardia | 98,381 | 33.4% |
| Angle Closure Glaucoma | 69,085 | 23.4% |

**SUPPLEMENTARY FIGURES**

**
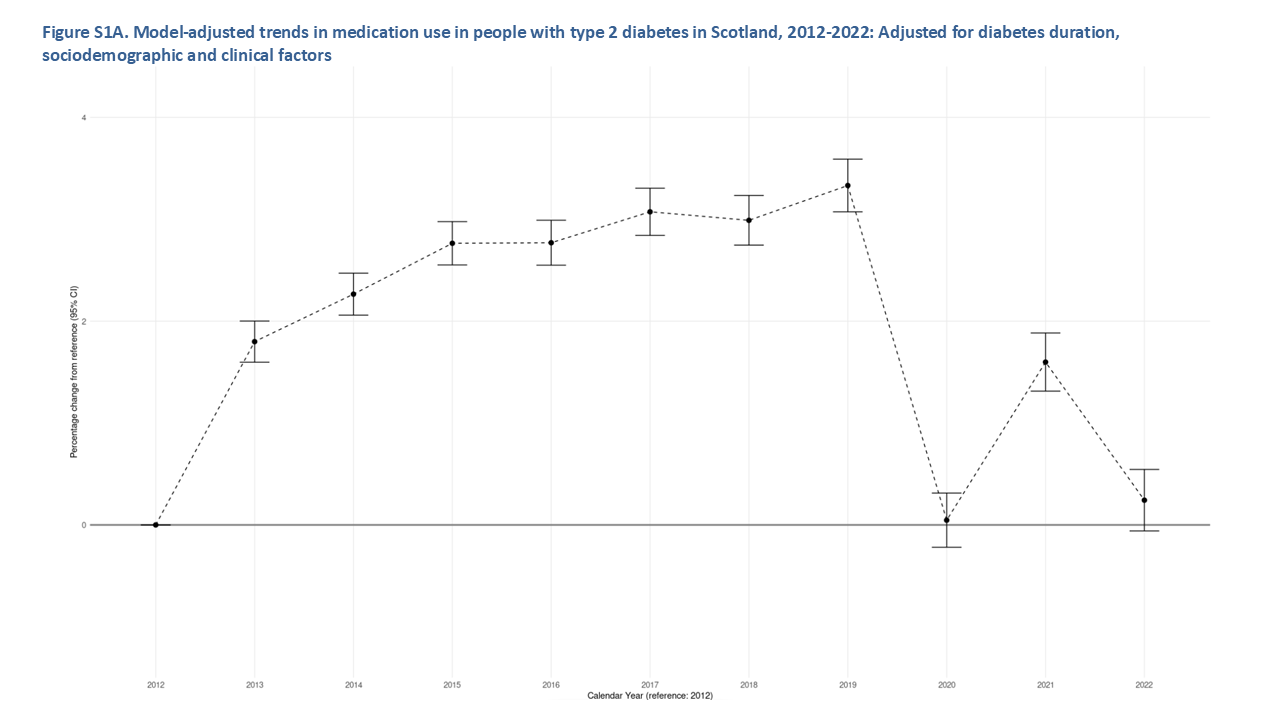
**

**
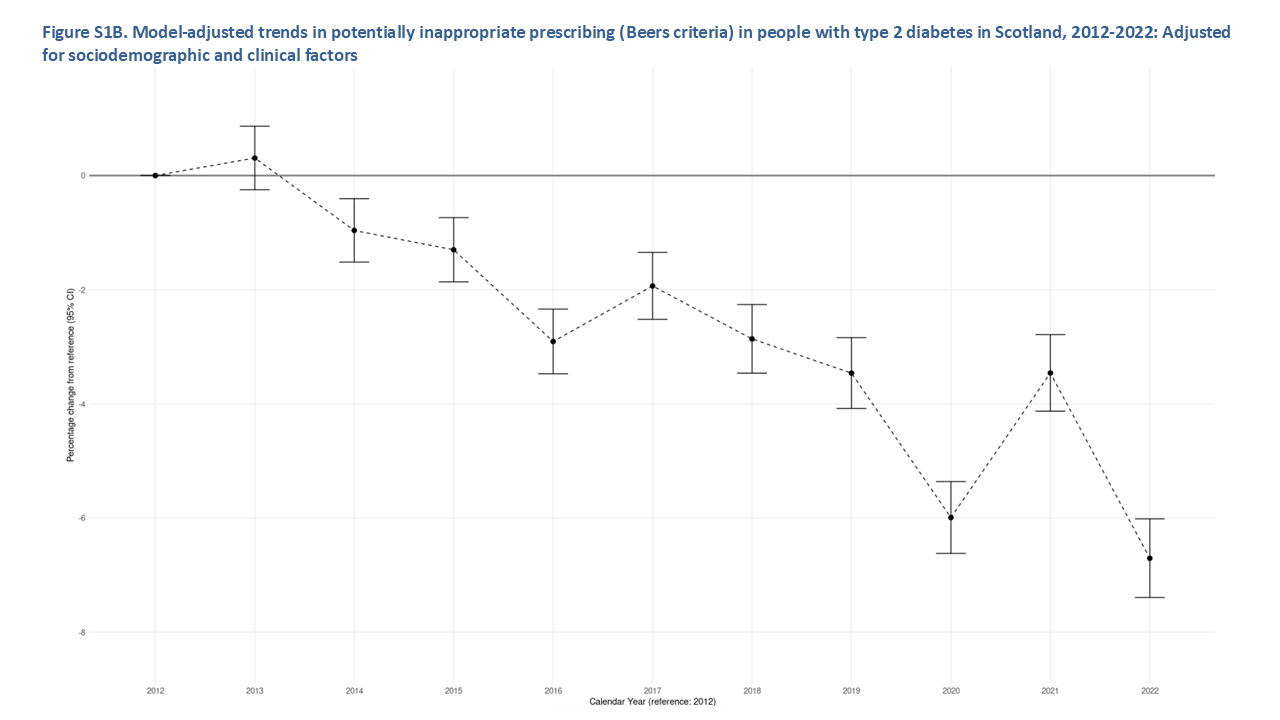
**

**
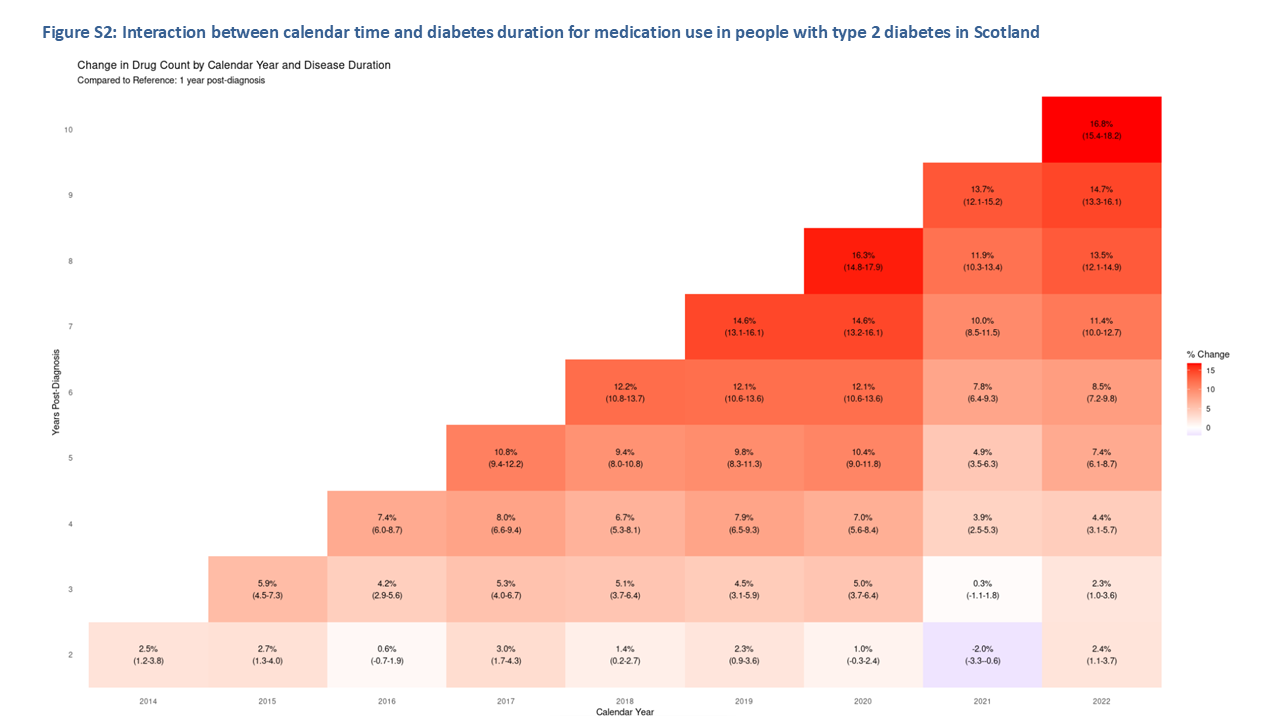
**

**
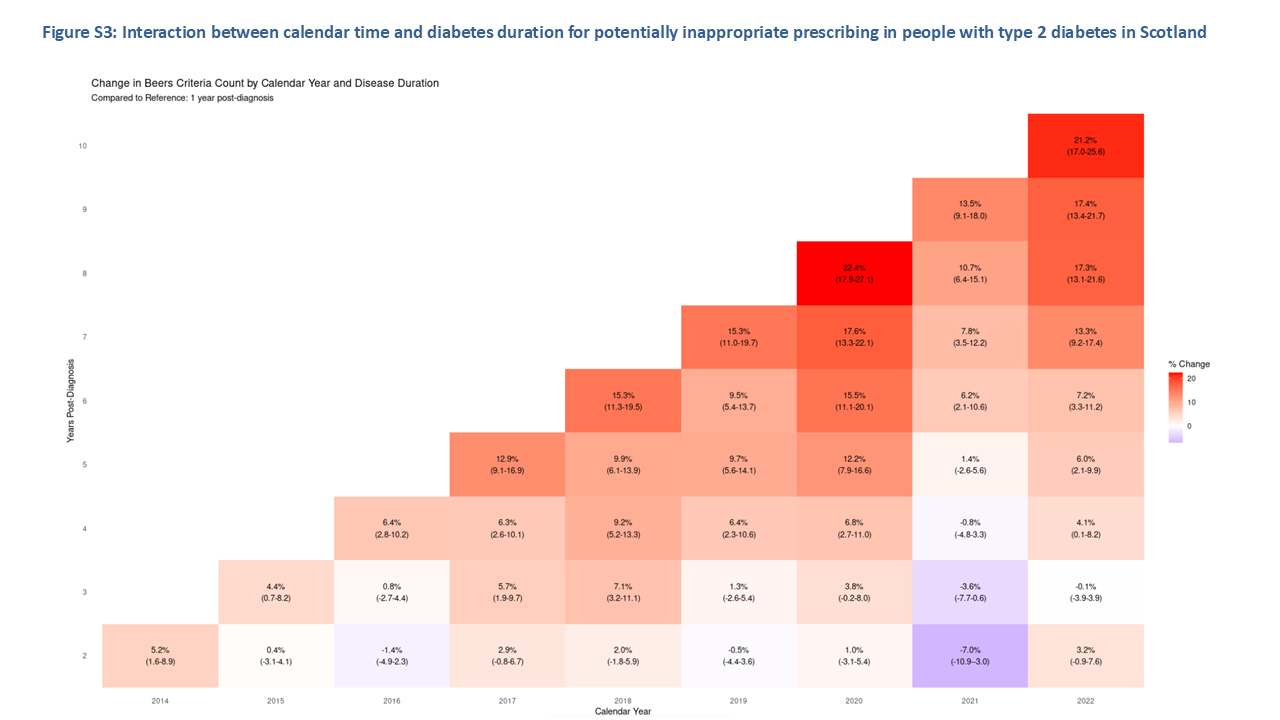
**

**
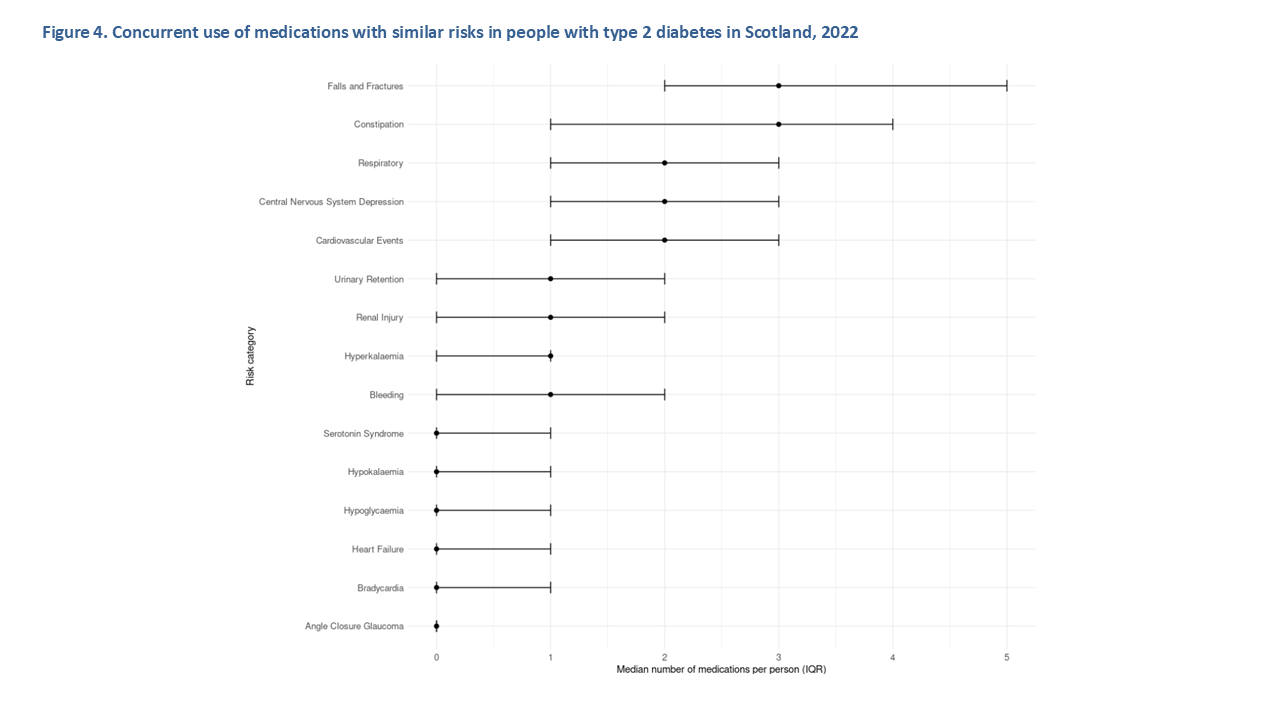
**
